# Type 2 innate lymphoid cells: at the cross-roads in allergic asthma

**DOI:** 10.1007/s00281-016-0556-2

**Published:** 2016-03-10

**Authors:** Leonie van Rijt, Helen von Richthofen, Ronald van Ree

**Affiliations:** 1 Department of Experimental Immunology, Academic Medical Center, University of Amsterdam, Room KO-104, Meibergdreef 9, 1105 AZ Amsterdam, The Netherlands; 2 Department of Otorhinolaryngology, Academic Medical Center, University of Amsterdam, Amsterdam, The Netherlands

**Keywords:** Type 2 innate lymphoid cells, Type 2 immune response, Allergic asthma

## Abstract

Allergic asthma is a chronic inflammatory disease of the lower airways that affects millions of people worldwide. Allergic asthma is a T helper 2 cell (Th2)-mediated disease, in which Th2 cytokines interleukin (IL)-4, IL-5, and IL-13 are closely associated with the symptoms. IL-4 is needed by B cells to switch toward an IgE response, IL-5 recruits and activates eosinophils while IL-13 increases mucus production. The identification of type 2 innate lymphoid cells (ILC2), which are able to rapidly produce large amounts of IL-5 and IL-13 in response to epithelial derived cytokines, implicated a new key player besides Th2 cells. ILCs constitute a family of innate lymphocytes distinct from T and B cells. ILC2s are located in various epithelial compartments in mice and human, including the lung. The recent finding of increased numbers of ILC2s in the airways of severe asthma patients prompts further research to clarify their immunological function. Murine studies have shown that ILC2s are an early innate source of IL-5 and IL-13 after allergen exposure, which induce airway eosinophilic infiltration, mucus hyperproduction, and airway hyperresponsiveness but not allergen-specific IgE production. ILC2s contribute to the initiation as well as to the maintenance of the adaptive type 2 immune response. Here, we review the recent progress on our understanding of the role of ILC2s in the immunopathology of allergic asthma, in particular by studies using murine models which have elucidated fundamental mechanisms by which ILC2s act.

## Introduction

Asthma is a chronic inflammatory airway disease that is characterized by airway hyperresponsiveness (AHR), eosinophilic inflammation, and mucus hyperproduction. Clinical symptoms include recurrent wheezing, coughing, and shortness of breath [1]. More than 300 million people worldwide are affected by asthma, making it one of the most common chronic diseases [2].

Th2 cells are the central players in allergic asthma, as they are major producers of the type 2 cytokines interleukin-4 (IL-4), IL-5, and IL-13, which are all tightly linked to the pathogenesis of asthma [3]. IL-4 induces IgE production by B cells, which can bind to high affinity Fcε receptors (FcεRI) on mast cells and basophils [4]. IL-5 activates eosinophils and attracts them to the lung, where they secrete numerous inflammatory cytokines and chemokines [5]. IL-13 directly affects the airway epithelium and smooth muscle cells in the lungs and thereby contributes to AHR, mucus hyperproduction, and, in persistent inflammation, to airway remodeling [6]. Current effective treatment of asthma is aimed at the suppression of symptoms by bronchodilators in combination with anti-inflammatory drugs, and only recently, this has been combined with therapies that target specific underlying immunological processes.

The key role for Th2 cells in driving allergies was challenged by the observation that mice with an absent adaptive immune system are still able to mount a strong type 2 response [7, 8]. This finding led to the identification of a previously unrecognized innate cell population, which was named type 2 innate lymphoid cell (ILC2) [9]. ILCs are classified into three different types, based on a similar cytokine profile corresponding to the helper T subsets Th1, Th2, and Th17, respectively. ILC2s lack the specific antigen receptor of Th2 cells, yet provide an important source of type 2 cytokines and are therefore potential new players in type 2 immunity and allergic asthma [10].

In this review, we will describe how ILC2s interact with the innate and adaptive immune system and thereby contribute to the immunopathology of allergic asthma. This cell population might be an attractive therapeutic target in the treatment of allergic asthma.

### IL-5 and IL-13 production by pulmonary ILC2s

Initial studies in mice used IL-25 or IL-33 inhalation to show that pulmonary ILC2s are capable of producing IL-5 and IL-13 after in vivo activation but secrete very little IL-4 [10–12]. These results were soon extended, as it was shown that pulmonary ILC2s have the capacity to produce large amounts of IL-5 and IL-13 after intranasal challenge with various allergenic substances, including ovalbumin (OVA) [10, 13], the protease papain [14, 15], house dust mite (HDM) [10], *Alternaria alternata* products [16, 17], glycolipid antigens [18], and the nonprotease allergen chitin, a polysaccharide from fungi, parasites and arthropods, known to induce Th2 cell-independent innate type 2 responses [19, 20]. In addition, IL-5 and IL-13 production by pulmonary ILC2s could be induced by influenza infection [21, 22] or pulmonary helminth infection [23]. Together, this indicates that ILC2s play an important role in immune responses to various intruding pathogens but are also involved in the aberrant immune response to allergens.

To determine the relevance of IL-5 and IL-13 production by ILC2s, their capacity to produce these cytokines should be compared to that of Th2 cells. After IL-25 inhalation, ILC2s constituted 50 % of IL-5^+^ cells and IL-13^+^ cells in the lung, and after IL-33 inhalation, this was even 80 % [10]. However, IL-25 or IL-33 treatment does not induce full T cell activation and might therefore overestimate the contribution of ILC2s to type 2 cytokine production [24]. Nevertheless, also in HDM- and OVA-induced asthma models, ILC2s produced a substantial part of the type 2 cytokines. After HDM challenge, the IL-5^+^ ILC2 population in the lung was approximately half the size of the IL-5^+^ Th2 cell population, and in the bronchoalveolar lavage fluid (BAL), these populations even had a similar size. Similar results were found when comparing IL-13^+^ ILC2s and IL-13^+^ Th2 cells after HDM challenge [10].

These results suggest that ILC2s contribute significantly to IL-5 and IL-13 production in murine models of allergic asthma. In support, papain treatment induced IL-5 and IL-13 secretion in lung explants from *Rag1* ^−/−^ mice (which have ILC2s but are deficient for T, B, and NKT cells), but not in lung explants from ILC-deficient *Rag2* ^−/−^ *Il2rg* ^−/−^ mice (which have no ILCs or other lymphocytes). Intracellular staining confirmed that the majority of IL-5^+^ and IL-13^+^ cells were indeed ILC2s [25]. The close proximity of lung ILC2s to medium-sized blood vessels explains how IL-5 could be released in the blood stream as a signal for the bone marrow to increase its eosinophil production [26].

### Understanding activation of ILC2s

ILC2s can be activated by various cytokines, and particularly epithelial cell-derived cytokines IL-25, IL-33, and TSLP have been shown to initiate ILC2 responses in both mice and humans [27]. These innate type 2 response-promoting cytokines can be released after inhalation of allergens. In addition, recently, other soluble factors have been reported to activate ILC2s, including prostaglandins [28–30] and leukotrienes [16]. Activation of ILC2s is defined by their expansion and type 2 cytokine production. Expansion of ILC2s can be achieved by recruitment of ILC2s to the site of challenge [22] as well as by in situ proliferation of resident cells [16, 17].

#### Epithelial cell-derived cytokines IL-25, IL-33, and TSLP

Mouse ILC2s can be activated by IL-25 [12, 31], IL-33 [18, 32–35], and TSLP [19, 25, 36], in vitro and in vivo. Recently, the main source of IL-33 and TSLP in response to chitin exposure was identified to be alveolar type II cells of the distal airways [20]. However, the relative importance of these cytokines in activating ILC2s is less clear and seems to vary in different locations and experimental settings. For example, a study by Neill et al. suggested that IL-25 is more potent than IL-33 in activating ILC2s in vivo, as ILC2 expansion in mesenteric lymph nodes was less impaired in *Ilrl1* ^−/−^ (IL-33R subunit) mice than in *Il17br* ^−/−^ (IL-25R subunit) mice in response to helminth infection [31]. Conversely, challenge of the same mouse strains with ragweed pollen or ovalbumin via the airways indicated that IL-33 is faster and more potent than IL-25 in promoting pulmonary ILC2 activation and AHR [37]. Chitin exposure induced contemporaneous expression of IL-33 and TSLP. The short coexpression of TSLP was shown to synergize with IL-33 to induce IL-5 and IL-13 production [20]. In contrast, Kim et al. found that skin ILC2s were independent of both IL-25 and IL-33 treatment, but instead relied on TSLP for their activation [36].

Studies in humans also suggest synergizing and varying roles for IL-25, IL-33, and TSLP in activating ILC2s. Mjösberg et al. found that ILC2s from human fetal gut or peripheral blood were activated in vitro in response to treatment with either IL-25 or IL-33, as long as it was combined with IL-2 treatment [38]. The same authors showed that TSLP alone could activate ILC2s from human peripheral blood in vitro by enhancing GATA-3 expression [39]. Conversely, Salimi et al. found that ILC2s from human skin were only activated directly by IL-33, whereas simultaneous treatment with IL-25 and TSLP could enhance IL-33-induced cytokine production in vitro [40]. This result corresponds to a study that investigated activation of mouse ILC2s in vitro, where IL-25 did not activate lung ILC2 directly, but only amplified the response of ILC2 that were activated by IL-33 plus IL-2, IL-7, or TSLP [25].

Interestingly, intranasal exposure to IL-25 evoked a second IL-13-producing ILC2 population (IL-17RB^+^/ST2^−^/CD127^lo^/KLRG1^hi^) which were called inflammatory ILC2s. This population was also induced after infection with *Nippostrongylus brasiliensis.* However, this increase was transiently and iILC2s seemed to act as a precursor for natural ILC2s, as they acquired expression of ST2 and responsiveness to IL-33 during ongoing inflammation. The *N. brasiliensis* induced iILC2s were also dependent on IL-25, as they were absent in IL-25 receptor (Il17rb^−/−^) knockout mice [41].

Together, these results indicate that IL-25, IL-33, and TSLP are all able to activate ILC2s, but that their relative contribution to this activation depends on the tissue, experimental setting, and state of disease. The redundancy of these cytokines in activating ILC2s has therapeutical implications, as targeting just one of these cytokines to inhibit ILC2s may not give satisfactory results. Indeed, studies in mice show that ILC2 activation in response to helminth infection [31] or *A. alternata* exposure [34] is only completely abolished in combined absence of IL-25 and IL-33 signaling. Moreover, ILC2 activation in response to chitin inhalation was only abolished in combined absence of IL-25, IL-33, and TSLP signaling [19].

#### Other cytokines: IL-2, IL-7, IL-9, and TL1A

In addition to epithelial cell-derived cytokines, other cytokines have been shown to enhance ILC2 activation. Several studies report that costimulation with IL-2 and/or IL-7 is required to activate ILC2 with epithelial cell-derived cytokines in vitro. For example, mouse ILC2 could only be activated by IL-33 in presence of IL-2 or IL-7 [21, 25]. Likewise, human ILC2s required presence of IL-2 to be activated by IL-25 or IL-33 [38]. In addition, studies report independent effects of IL-2 and IL-7. ILC2s that were isolated from fat-associated lymphoid clusters (FALCs) proliferated in response to IL-2 treatment in vitro [32]. Culturing lung ILC2s on OP9 stromal cells with IL-2 or IL-7 supported the survival but not expansion of ILC2s [25]. However, although the requirement for IL-7 signaling in ILC2 development is well established, its role in ILC2 activation in vivo remains to be confirmed. It was recently demonstrated that IL-2 might activate ILC2s in vivo, as injection of *Rag1* ^−/−^ mice (which lack T, B, and NKT cells but do have ILC2s) with IL-2-anti-IL-2 antibody complexes (which facilitate slow release of IL-2) increased ILC2 numbers in the lung, mesenteric LN, spleen, and skin [13, 42, 43]. IL-2-induced ILC2 expansion also correlated with efficient worm expulsion [13]. The responsiveness to IL-2 has interesting implications, as T cells are the main source of IL-2 in vivo and might therefore regulate ILC2 activation and survival [42]. Indeed, ILC2s were able to expand in *N. brasiliensis*-infected *Rag2* ^−/−^ mice (which lack T, B and NKT cells but do have ILC2s), but their numbers could not be maintained [31]. A number of non-T cell sources for IL-2 have been reported including dendritic cells, eosinophils and ILC3 (IL-17-producing ILCs) [43].

Another cytokine that was reported to enhance ILC2 function is IL-9 [14, 20, 23]. Blocking IL-9 signaling with neutralizing antibodies [14] or by using *Il9r* ^−/−^ mice [23] reduced ILC2 activation in response to papain inhalation [14] or *N. brasiliensis* infection [23]. In addition, IL-9 induced expression of the antiapoptotic protein BCL3 on ILC2s in *N. brasiliensis*-infected lungs, and thereby promoted their survival [23]. With the use of IL-9 reporter mice, it was shown that ILC2s themselves provided the major source of IL-9, indicating that IL-9 acts on ILC2s in an autocrine fashion [14, 23]. Furthermore, IL-9 expression in response to papain treatment was strongly decreased in *Rag1* ^−/−^ mice compared to WT mice, and this effect was rescued by application of IL-2, suggesting that IL-9 production by ILC2s is dependent on IL-2 provided by the adaptive immune system [14]. IL-9 was also induced in response to chitin, resulting in an increased IL-5 and IL-13 production. The IL-9 induction was dependent on the induction of the transcription factor interferon regulatory factor 4 [20].

Recently, two studies showed that the TNF-like ligand 1A (TL1A) can activate ILC2s [44, 45]. TL1A was discovered as a costimulatory cytokine that promotes T cell activation by binding to Death receptor 3 (DR3) [46]. Both mouse and human ILC2s constitutively express DR3 [44, 45]. Yu et al. showed that TL1A alone could activate mouse ILC2s in vitro as well as in in vivo, whereas Meylan et al. found that TL1A alone could only activate ILC2s in vitro. However, both studies showed that TL1A signaling enhanced IL-33- and IL-25-induced ILC2 activation in mice, in vivo [44, 45]. The same enhancing effect on IL-33 or IL-25 treatment was found for ILC2s from human peripheral blood [44]. Together, this suggests that TL1A can act as an ILC2 costimulator. Interestingly, TL1A is produced by DCs and macrophages in response to TLR activation or Fc receptor crosslinking [46], indicating that ILC2 activation is also regulated by innate immune cells.

#### Leukotrienes and prostaglandins

Recently, it was shown that ILC2s can be activated by certain leukotrienes and prostaglandins. These lipid mediators are associated with allergic type 2 responses and are produced by various activated innate immune cells, including mast cells, eosinophils, macrophages, and dendritic cells [47].

Doherty et al. showed that lung and bone marrow ILC2s from naive and *Alternaria*-challenged mice expressed the cysteinyl leukotriene receptor 1 (CysLT1R). Cysteinyl leukotrienes have been associated with allergic asthma in mice [48] and humans [49]. Treatment with leukotriene D_4_ (LTD_4_), a CysLT1R ligand, activated ILC2s in vitro and in vivo, and this response could be blocked by treatment with the CysLT1R antagonist montelukast, which is used in treatment of allergic asthma [16].

In humans, expression of CRTH2 is commonly used to identify ILC2s [38]. Prostaglandin D_2_ (PGD_2_), a CRTH2 ligand, is highly expressed in human mucosal sites and is known for its chemotactic effects on Th2 cells [50]. It was therefore hypothesized that PGD_2_ may also recruit ILC2s to mucosal tissue [29]. Indeed, PGD_2_ treatment induced chemotaxis and cytokine production by human ILC2s from peripheral blood [29, 30] and skin [28], in vitro. This response could be prevented by treatment with CRTH2 antagonists [28–30]. Mast cells provide a major source of PGD_2_ during allergic responses, and ILC2s could be activated by supernatant from activated human mast cells, but not in the presence of a CRTH2 antagonist [28].

Barnig et al. identified lipoxin A_4_ (LXA_4_) as a negative regulator of ILC2s [30]. Human ILC2s from peripheral blood expressed the LXA_4_ receptor ALX/FPR2, and treatment with LXA_4_ decreased cytokine production by activated ILC2s in vitro [30]. The natural generation of LXA_4_ is decreased in severe asthma and implicates unrestrained activation of ILC2s. This interaction might contribute to the function of LXA_4_ as a natural pro-resolving mediator in allergic asthma [51].

## ILC2s in allergic asthma in mice

**Fig. 1:**
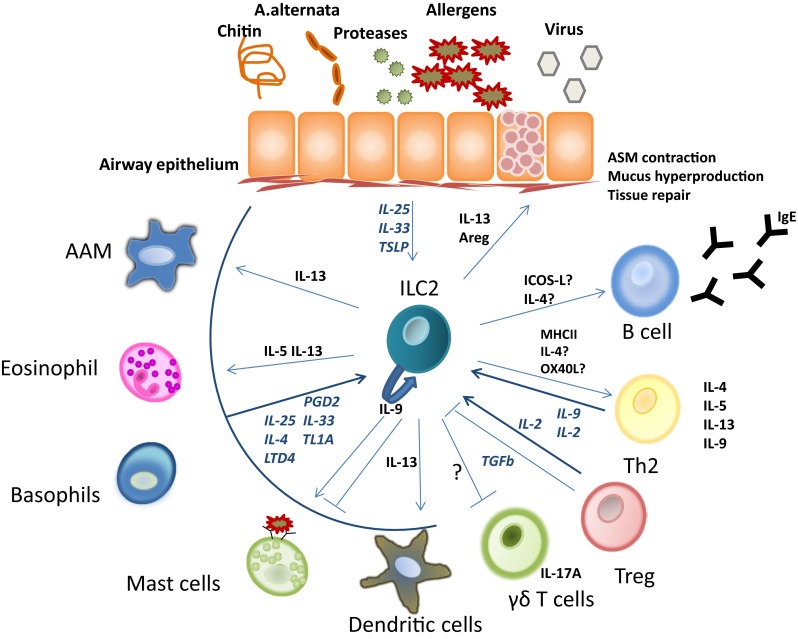
ILC2 interactions in allergic asthma. The inbetweeners: ILC2s in the immunopathology of allergic asthma. ILC2s respond to IL-25, IL-33, or TSLP, which is produced by airway epithelial cells in response to allergen encounter or infection with helminthes or viruses. In addition, ILC2s may be activated by innate cell-derived PGD_2_, LTD_4_, TL1A, IL-25, or IL-33. IL-9 activates ILC2s in an autocrine manner. The production of IL-5 and IL-13 by ILC2s contributes to typical allergic asthma hallmarks, including pulmonary eosinophilia, mucus hyperproduction, and AHR. ILC2-derived IL-13 was shown to induce accumulation of alternatively activated macrophages and promote migration of DCs from the lung to lymph node. ILC2s also interact with T cells, likely via MHCII expression and possibly via expression of IL-4 and OX40L. In turn, T cells can activate ILC2s by secreting IL-2. In addition, ILC2s may interact with B cells, possibly via expression of ICOS-ligand or IL-4. The presence of ILC2s might also have beneficial effects in allergic asthma, as their production of amphiregulin (Areg) was shown to promote tissue repair of the epithelium in pulmonary inflammation

ILC2s produce several type 2 cytokines and can thereby affect various cells that are involved in the pathogenesis of allergic asthma (Fig. 1). ILC2-deficient murine models of allergic asthma have provided an important tool in elucidating these interactions, in which comparison of *Rag2* ^−/−^ mice (deficient in T, B, and NKT cells) with *Rag2* ^−/−^ *Il2rg* ^−/−^ mice (deficient in T, B, NKT, and ILCs) allowed for the investigation of ILC2 effector functions independent of the adaptive immune system, whereas RORα-deficient staggerer (*Rora* ^sg/sg^) mice provide a model to investigate ILC2 function in the context of the adaptive immune system. RORα is an important transcription factor in ILC2s. RORα^sg/sg^ mice suffer from severe neurological defects from birth; therefore, mainly bone marrow transplanted mice (BMT) are used to prevent neural abnormalities. Transplantation of RORα^sg/sg^ BM in an irradiated host provides a normal hematological repopulation, except that BMT mice lack the IL-25 induced expansion of ILC2s [52].

### Interaction of ILC2s with innate immune cells and structural cells

#### ILC2s contribute to pulmonary eosinophilia

IL-5 is essential for eosinophil development and activation [53], and therefore, ILC2-derived IL-5 is likely to affect eosinophils. Indeed, several studies suggest that activated ILC2s contribute to eosinophilic inflammation. For example, Doherty et al. showed that intranasal *Alternaria* or LTD_4_ administration induced pulmonary eosinophilia in *Rag2* ^−/−^ mice (lack T, B, and NKT cells but do have ILC2s), but not in *Il7ra* ^*−/−*^ mice, which lack all ILCs in addition to B and T cells [16, 17]. Likewise, Halim et al. showed that intranasal papain treatment increased the number of eosinophils in the lungs of WT and *Rag1* ^−/−^ mice, but not in ILC-deficient *Rag2* ^−/−^ *Il2rg* ^−/−^ mice. Furthermore, depletion of ILC2s in *Rag1* ^−/−^ mice with neutralizing CD25 antibodies decreased papain-induced eosinophilia, whereas adoptive transfer of ILC2s into *Rag2* ^−/−^ *Il2rg* ^−/−^ enhanced papain-induced eosinophilia [25]. These studies indicate that activated ILC2s induce pulmonary eosinophilia independent of the adaptive immune system.

In a subsequent study, Halim et al. investigated whether ILC2s affect eosinophilia in the presence of Th2 cells. Adoptive transfer of WT bone marrow cells into *Rag2* ^−/−^ *Il2rg* ^−/−^ mice restored papain-induced and IL-25-induced pulmonary eosinophilia, whereas adoptive transfer of ILC2-deficient *Rora* ^sg/sg^ bone marrow cells did not. The presence of other pulmonary leukocytes, including neutrophils, macrophages, DCs, NK cells, NKT cells, T cells, and B cells, was not differentially affected in papain-treated mice that had received WT bone marrow cells versus mice that had received *Rora* ^sg/sg^ bone marrow cells [54]. Additionally, in an influenza A virus (IAV)-induced pulmonary inflammation model, in *vivo* depletion of ILC2s and T cells with a neutralizing anti-Thy1.2 antibody in WT mice reduced eosinophil numbers in the lung, which could be rescued by the adoptive transfer of total Thy1.1^+^ cells (which include ILC2 and T cells) but not CD3^+^ Thy1.1^+^ lung cells (which only include T cells) [22].

Together, these results indicate that ILC2s contribute to eosinophilia in mouse models of allergic asthma, both in the absence and presence of the adaptive immune system. It seems likely that this effect is mediated by ILC2-derived IL-5, yet no study has directly assessed this. The only indication that ILC2-derived IL-5 is sufficient to induce eosinophilia came from a recent study that used IL-5 reporter (Red5) mice to investigate pulmonary IL-5 production [19]. Red5 mice express a tdTomato(RFP)-IRES-Cre replacement allele at the translation start site of the endogenous *Il5* gene, resulting in production of RFP instead of IL-5 upon commitment to *Il5* translation [55]. *Il5* ^red/red^ mice (which are IL-5 deficient) had strongly diminished numbers of eosinophils in the lungs after repeated chitin inhalation, whereas ILC2 numbers were not affected. Strikingly, RFP expression was found exclusively in ILC2s [19], indicating that ILC2-derived IL-5 is critically required for chitin-induced eosinophilia. In addition, ILC2-derived IL-13 may also contribute to allergen-induced pulmonary eosinophilia. Chitin-treated *Il13* ^*−/−*^ *Il4* ^*−/−*^ mice had reduced eosinophil numbers in the lung, whereas *Il4* ^*−/−*^ mice had not. The source of IL-13 after challenge with chitin was confined to ILC2s as elucidated by IL-13 reporter mice (*Il13* ^smart/smart^) [19]. In support, Barlow et al. found that IL-25-induced eosinophilia in the BAL was decreased in *Il13* ^−/−^ mice compared to WT mice, which could partly be rescued by the adoptive transfer of WT ILC2, but not by transfer of *Il13* ^−/−^ deficient ILC2 [56]. Together, these reports suggest that ILC2-derived IL-5 and IL-13 contribute nonredundantly to eosinophilia. This corresponds with earlier findings on allergen-induced eosinophilia, which show that IL-5 promotes development and survival of eosinophils, whereas IL-13 mediates recruitment of eosinophils through secretion of eotaxins [57]. In line with this, Van Dyken et al. found that chitin challenge increased expression of eotaxin-1 in the lungs and that chitin-induced eosinophilia was decreased in mice lacking the eotaxin-1 receptor CCR3 [19].

#### ILC2s contribute to AHR and mucus hyperproduction

IL-13 acts directly on airway epithelial cells and smooth muscle cells and thereby induces AHR and mucus hyperproduction in allergic asthma [6]. As ILC2s are capable of producing IL-13 in response to allergen exposure, it is proposed that ILC2s also contribute to the pathogenesis of these asthma symptoms.

This hypothesis was supported by an influenza (H3N1)-induced pulmonary inflammation study. H3N1 infection induced AHR in *Rag2* ^−/−^ mice, which was abolished after ILC2 depletion with a neutralizing Thy1.2 antibody. In addition, H3N1 infection failed to induce AHR in *Il13* ^−/−^ mice, which was restored by adoptive transfer of WT ILC2s [21]. ILC2-derived IL-13 also affected AHR in cytokine- and allergen-based mouse models of pulmonary inflammation. Kim et al. found that *Il13* ^−/−^ mice showed reduced AHR after IL-33- or glycolipid (α-Galcer) inhalation compared to WT mice, which could be rescued by adoptive transfer of WT ILC2s [18]. Barlow et al. found similar results for IL-25-induced AHR, and importantly, they showed that adoptive transfer of WT ILC2s, but not *Il13* ^−/−^ ILC2s, could rescue IL-25-induced AHR in *Il13* ^−/−^ mice [56]. This indicates that ILC2-derived IL-13 is sufficient to induce AHR in response to IL-25 inhalation. However, the contribution of ILC2-derived IL-13 to allergen-induced AHR within the context of other IL-13-producing cells remains to be determined.

Besides the involvement in AHR, some studies suggest that ILC2s also contribute to mucus hyperproduction in allergic asthma models. For example, papain inhalation induced mucus hyperproduction in the lungs of WT and *Rag1* ^−/−^ mice, but not in the lungs of *Rag2* ^−/−^ *Il2rg* ^−/−^ mice [25]. Furthermore, adoptive transfer of WT bone marrow cells, but not *Rora* ^sg/sg^ bone marrow cells, restored papain-induced mucus hyperproduction in *Rag2* ^−/−^ *Il2rg* ^−/−^ mice [54]. These studies are indicative for an effect of ILC2s on mucus hyperproduction, yet they do not prove that this effect is mediated via IL-13 secretion.

#### Interactions with macrophages, γδ T cells, ILC3, mast cells, and basophils

Although these interactions are less well established, it appears that ILC2s also interact with other innate immune cells that are involved in allergic asthma.

It was recently suggested that ILC2s may contribute to allergen-induced accumulation of alternatively activated macrophages (AAMs, also known as M2 macrophages) [19]. IL-4 and IL-13 stimulate differentiation into AAMs, which typically express arginase-1 and chitinases [58]. AAMs are generally considered to be anti-inflammatory but are also implied to play a role in pathogenic type 2 responses [59]. The study that investigated chitin-induced eosinophilia found that chitin-challenged *ll13* ^*−/−*^ *Il4* ^*−/−*^ mice had reduced AAM numbers in the lung, whereas *Il4* ^*−/−*^ mice had not. IL-13 was exclusively expressed by ILC2s, which suggested that ILC2-derived IL-13 contributed to chitin-induced AAM accumulation [19].

The same authors crossed *Il5* ^Red5/Red5^ or *Il13* ^YetCre/YetCre^ mice with mice that carry a *Gt(Rosa)26* ^DTA^ allele, to create a mouse model in which cells get killed by diphtheria toxin α as soon as they commit to IL-5 or IL-13 production, respectively. Challenging these mice with chitin led to deletion of ILC2s and a reduction in AAM accumulation and eosinophilia, which supports earlier findings [19]. Surprisingly, ILC2 deletion also increased the levels of several inflammatory cytokines including TNFα, IL-1β, and IL-23 and increased the activation of IL-17A-producing γδ T cells, which caused an increase in airway neutrophilia. Intriguingly, chitin-treated IL-5- and IL-13-deficient mice had similar numbers of γδ T cells and neutrophils as WT controls, suggesting that the suppressive effects of ILC2s on γδ T cells and neutrophils were not mediated via IL-5 or IL-13 signaling [19].

Two recent reports showed colocalization of ILC2s and mast cells, suggesting an interaction between these cells. Barnig et al. found ILC2s in the close proximity of mast cells in the human lung [30]. Roediger et al. used intravital multiphoton microscopy to track the movement of ILC2s in the skin of healthy mice and found that ILC2s specifically interacted with mast cells. Interestingly, these ILC2s constitutively expressed IL-13, and in vitro experiments showed that IL-13 suppressed the release of TNFα and IL-6 by mast cells, suggesting an inhibitory effect of ILC2s on mast cells in this context [42]. Mast cells and ILC2s might also interact via mast cell-derived PGD_2_ and LTD_4_, which is released upon allergen challenge-induced FcεRI cross-linking and might recruit and activate ILC2s [16, 28–30]. However, this remains to be confirmed in vivo and in the context of allergic asthma. Roy et al. reported that mast cell chymase has the ability to degrade IL-33, indicating that mast cells can have a regulatory function in the balance of IL-33, implicating a feedback mechanism on the activation of ILC2s [60].

One recent study suggested interactions between ILC2s and basophils, as clusters of ILC2s and basophils were found in inflamed human and murine skin. The presence of basophils preceded the recruitment of ILC2s to atopic dermatitis-like skin lesions in mice. Interestingly, basophil-derived IL-4 was required for the optimal recruitment of ILC2s to these skin lesions, and ILC2s expressed the IL-4 receptor, suggesting a novel type of ILC2 regulation [61]. However, it remains to be established whether this applies to ILC2s at other locations and whether ILC2s are also able to affect basophils. Furthermore, depletion of basophils in a papain-induced pulmonary inflammation model had no effect on pulmonary eosinophilia, which argues against a major role of basophils in pulmonary inflammation and ILC2 regulation [62].

### Interaction of ILC2s with the adaptive immune system

ILC2 research has focused on the ability of ILC2s to secrete type 2 cytokines and thereby enhance the activity of innate immune cells (e.g., eosinophils) in allergic asthma. Interestingly, recent studies suggest that ILC2s may not only affect innate cells but also contribute to the activity of adaptive immune cells (e.g., Th2 cells) in allergic asthma. For example, Gold et al. investigated the contribution of ILC2s to the adaptive immune response to HDM. ILC2-deficient *Rora* ^sg/sg^ BMT mice (irradiated WT host mice transplanted with *Rora* ^sg/sg^ BM) showed impaired leukocyte infiltration in the lungs, as well as reduced serum levels of HDM-specific IgE, compared to WT BMT mice after HDM exposure [63]. Th2 cells amplify allergic inflammation, and therefore ILC2-mediated activation of Th2 cells may have large implications for the overall contribution of ILC2s to allergic asthma.

#### ILC2s affect initiation of the adaptive immune response via DCs

ILC2s might affect the adaptive immune response by influencing Th2 cell differentiation (possibly via DCs), and/or by influencing the activity of primed Th2 cells or IgE-producing B cells. This question was assessed by Halim et al., who compared papain-induced Th2 cell generation in the mediastinal LN (mLN) of WT BMT mice versus *Rora* ^sg/sg^ BMT mice [64]. Via intracellular cytokine staining, they showed that Th2 cell differentiation occurred 6 days after papain treatment in the mLN of WT mice, and that this response was impaired in *Rora* ^sg/sg^ BMT mice compared to WT BMT mice. Similar results were found in HDM- or fungal protease-allergen-treated mice. Notably, the adoptive transfer of WT ILC2s into papain-treated *Rora* ^sg/sg^ BMT mice restored the number of Th2 cells [64]. Together, these results suggested that ILC2s are involved in driving Th2 cell differentiation in response to allergen encounter.

Although it has been shown that ILC2s accumulate in the mLN during airway inflammation, it remains the question whether they play a direct role in skewing naive T cells toward Th2 differentiation. As IL-4 plays a critical role in Th2 cell differentiation [65], it was therefore investigated whether ILC2s might promote Th2 cell differentiation via IL-4. Some studies report that pulmonary ILC2s have the ability to produce small amounts of IL-4 after in vivo activation with IL-25 or IL-33 [10], OVA[10], papain [14], and HDM [10, 66], as measured by ELISA or intracellular FACS staining. IL-4 production by ILC2s may depend on specific activating stimuli, as LTD_4_, as shown in vitro. In contrast, IL-33 was not able to induce IL-4 production by ILC2s in vitro [16]. It remains to be determined whether ILC2s also produce IL-4 in the close proximity of naïve CD4^+^ T cells in vivo.

However, differentiation toward Th2 cells can occur in an IL-4-independent pathway, as shown by the induction of Th2 cells in IL-4-deficient mice after *N. brasiliensis* infection [67]. In concordance, Halim et al. found that papain-challenged *Il4* ^−/−^ mice displayed a normal Th2 response. Interestingly, they found that papain-treated *Il13* ^−/−^ mice had strongly decreased Th2 cell numbers in the mLN compared to WT mice. Intracellular cytokine staining showed that ILC2s were the predominant source of IL-13 after papain treatment, suggesting that ILC2-derived IL-13 promoted Th2 cell differentiation. This was confirmed in a transgenic TCR model, in which CFSE-labeled OVA-specific OT-II T cells were injected into WT BMT mice or *Rora* ^sg/sg^ BMT mice. OVA plus papain treatment induced normal T cell proliferation but failed to induce Th2 cell differentiation in *Rora* ^sg/sg^ BMT mice. This effect could be rescued by adoptive transfer of WT ILC2, but not by *Il13* ^−/−^ ILC2, indicating that ILC2-derived IL-13 is required for papain-induced Th2 differentiation. Notably, the IL-13 receptor was present on DCs but not on CD4^+^ T cells, suggesting that this effect was mediated via DCs. Indeed, DCs with CD40^+^ expression (a surface marker that is implied to induce Th2 differentiation) were present at normal numbers in the lungs but strongly decreased in the mLN of *Rora* ^sg/sg^ BMT mice, which could be rescued by IL-13 injections. Subsequent in vitro migration assays showed that IL-13 increased the capacity of DCs to migrate toward a CCL21 gradient, which is highly expressed in the lymph node [64].

Together, these results indicate that ILC2s might facilitate Th2 differentiation via inducing efficient migration of activated DCs from the lung to the mLN [64]. The question remains whether ILC2s might skew DCs toward a pro-Th2 DC phenotype, e.g., expression of OX40L, ICOS-L, Notch ligand Jagged1, and secreting cytokine IL-6 but not IL-12.

#### Interactions between ILC2s and CD4^+^ T cells

In addition to an indirect, DC-mediated interaction, recent studies suggest that pulmonary ILC2s and CD4^+^ T cells might interact directly with each other [68, 69]. In vitro studies suggested that CD4^+^ T cells can activate ILC2s via IL-2 signaling, which corresponds with earlier findings of IL-2-mediated activation of ILC2s [32, 42]. Wilhelm et al. demonstrated that IL-2 treatment enhanced the autocrine IL-9 production by ILC2s, resulting in increased IL-5 and IL-13 [14]. In contrast, Mirchandani did not find an increased IL-9 production after IL-2 treatment [68], suggesting that IL-2 can have distinct stimulating effects on ILC2s in different settings.

On the other hand, the presence of ILC2s also enhanced anti-CD3/anti-CD28-induced CD4^+^ T cell proliferation [69] and type 2 cytokine production [68, 69], whereas IFN-γ production remained constant [69] or even decreased [68]. ILC2s and CD4^+^ T cells likely interacted in a cell contact-dependent manner, because separation of CD4^+^ T cells and ILC2s in a transwell system strongly decreased ILC2-dependent CD4^+^ T cells activation [68]. Drake et al. showed that increased type 2 cytokine production by cocultured ILC2s and CD4^+^ T cells was partly dependent on OX40L expression by ILC2s, likely through interaction with OX40 on CD4^+^ T cells [69]. OX40L expression by DCs has been shown before to drive Th2 cell differentiation [70]. In addition, Drake et al. found that ILC2-derived IL-4 might play a role in CD4^+^ T cell activation, since coculturing of *Il4* ^−/−^ ILC2s with WT CD4^+^ T cells strongly diminished total IL-5 and IL-13 production as compared to coculturing WT ILC2s and CD4^+^ T cells [69]. However, it should be noted that the authors measured total cytokine production by cocultured ILC2s and CD4^+^ T cells and did not formally prove which cell type was the source of these cytokines [69]. In contrast, Mirchandani et al. found that ILC2-mediated activation of CD4^+^ T cells was independent of OX40- and IL-4 signaling [68]. Instead, their results suggested that ILC2s might interact with CD4^+^ T cells by presenting antigens on MHCII, as OVA-peptide-pulsed ILC2s could induce proliferation of OVA-specific transgenic DO11.10 T cells, which was blocked by adding a neutralizing MHCII antibody [68].

The potential capacity of ILC2s to present antigens was demonstrated by Oliphant et al. [13]. ILC2s that were isolated from murine mesenteric lymph nodes expressed MHCII, CD80, and CD86. Furthermore, they were able to endocytose and process antigen in vitro, albeit at a much lower efficiency than DCs and B cells, but could not induce proliferation of OVA-specific OT-II transgenic (OTII Tg) T cells after coculture unless OVA peptide was added [13]. The proliferation was abolished after using a neutralizing MHCII antibody or by using *MhcII* ^−/−^ ILC2s but also after combined treatment with neutralizing CD80 and CD86 antibodies [13]. ILC2s may provide T cells with costimulation on CD28 [13]. The capacity to present allergens in vivo awaits confirmation and in comparison to other APCs, like DCs. Additionally, the capacity to present antigens may be specific for certain ILC2 subsets. For example, mLN ILC2s had substantially higher MHCII expression than bronchoalveolar and lung ILC2s [13]. Coculturing of peptide-pulsed ILC2s also strongly enhanced ILC2 proliferation and type 2 cytokine production, as compared to controls lacking peptide. Interestingly, this effect was blocked by treatment with neutralizing antibodies against MHCII, CD80, CD86, or IL-2 [13]. These results implied that ILC2-expressed MHCII, CD80, and CD86 were not only required for ILC2-mediated activation of CD4^+^ T cells but also for activation of ILC2s themselves, possibly by stimulating CD4^+^ T cells to provide them with IL-2.

To investigate reciprocal activation between ILC2s and CD4^+^ T cells in vivo, cotransfer experiments were performed in murine models of allergic asthma [68, 69]. *Il7ra* ^−/−^ mice (which are deficient in ILCs and T cells) were reconstituted with ILC2 and/or CD4^+^ T cells from naive mice, and subsequently treated with a combination of OVA and a cysteine protease (bromelain)[69]. OVA plus bromelain inhalation induced a small increase in pulmonary eosinophilia and IL-13 levels in the BAL of mice that had been reconstituted with ILC2s or CD4^+^ T cells, whereas a large increase was found in mice that had been reconstituted with ILC2s and CD4^+^ T cells. Remarkably, the latter increase was larger than the additive effect of ILC2- and CD4^+^ T cell-transfer alone, which suggested a synergistic effect [69]. Similar enhancing effects of ILC2s on CD4^+^ T cell proliferation and type 2 cytokine production were found by Mirchandani et al., who cotransferred ILC2s and DO11.10 CD4^+^ T cells in OVA plus IL-33-treated ST2-deficient mice [68]. However, for both studies, it cannot be excluded that interactions between ILC2s and CD4^+^ T cells in vivo were mediated via another cell type, for example DCs. A potential direct interaction between CD4^+^ T cells and ILC2 in vivo was assessed by Oliphant et al., who tested the requirement for MHCII expression by ILC2 for expulsion of intestinal *N. brasiliensis* in *Il13* ^−/−^ mice [13]. *Il13* ^−/−^ mice showed delayed helminth expulsion, which could be rescued by adoptive transfer of WT ILC2s, but not *MhcII* ^−/−^ ILC2s. However, no difference in CD4^+^ T cell numbers could be observed in mice that had received WT ILC2 versus *MhcII* ^−/−^ ILC2 [13]. This implied that ILC2-expressed MHCII was required for efficient helminth expulsion, yet not for proliferation of CD4^+^ T cells in vivo. Possibly ILC2s were dependent on MHCII expression to receive activating signals from CD4^+^ T cells (e.g., IL-2).

Although ILC2s contribute to the initiation of Th2 cell differentiation, they do not seem to be required for the activation of memory Th2 cells. ILC2 deficient mice were capable of eliciting a full-blown airway inflammation, when initial Th2 cell differentiation was induced by an i.p. injection with OVA with the adjuvant alum [63].

Recently, it was described that regulatory T cells might also regulate activation of ILC2s besides their recognized suppressive effect on effector Th2 cells. In a self-limited OVA-induced allergic airway inflammation, it was demonstrated that the de novo generation of regulatory T cells coincided with a decrease in IL-13 production by ILC2s in a TGF-β-dependent manner [71]. Interestingly, the persistence of airway inflammation, airway hyperreactivity and remodeling was demonstrated to be dependent on ILC2s rather than on antigen specific T cells in a chronic asthma model [72].

#### Interactions between ILC2s and B cells

One of the initial reports that characterized ILC2s described interactions between ILC2s and B cells. Coculturing of ILC2s from the FALC with splenic B cells enhanced IgA production. Furthermore, ILC2s enhanced proliferation of peritoneal B1 cells but not B2 cells, which was dependent on ILC2-derived IL-5. In addition, B1 cells that were adoptively transferred into *Rag2* ^−/−^ mice proliferated more than when they were transferred into *Rag2* ^−/−^ *Il2rg* ^−/−^ mice, and cotransfer of B1 cells with ILC2 but not CD4^+^ T cells into *Rag2* ^−/−^ *Il2rg* ^−/−^ mice induced B1 cell proliferation [32]. Therefore, the authors suggested that ILC2s were able to activate B1 cells in vivo. However, these results have not been replicated and might not be relevant for the role of ILC2s in allergic asthma.

Recently, a novel Thy-1^+^ Sca-1^+^ ILC2-like cell type was described in the murine spleen that did not express c-Kit, IL-7R, or ST2, but instead expressed IL-18R. These ILC2-like cells proliferated and produced IL-5 and IL-13 after in vitro stimulation with IL-2 plus IL-18, or after coculture with B cells. Interestingly, coculturing these ILC2-like cells with anti-CD40 plus IL-4-treated B cells strongly enhanced IgE production. This effect was abolished when the cell populations were separated in a transwell system, suggesting a contact-dependent interaction [73].

An interaction between ILC2s and B cells might be mediated via the costimulatory molecule ICOS, that is highly expressed by ILC2s and may interact with ICOS-ligand-expressing B cells [31]. Interestingly, ILC2s also express ICOS-L and provide self-stimulation, important for survival and cytokine production. The ICOS-ICOS-L interaction was demonstrated to be required for murine and human ILC2 mediated induction of airway inflammation and hyperreactivity [74]. However, these interactions as well as their potential consequences for B cell function and type 2 responses warrant further studies.

## ILC2s in human allergic asthma

The earliest association between ILC2s and human asthma was probably described in 2009 by Allakhverdi et al., although ILC2s were not yet characterized at that time [75]. The authors found CD34^+^ non-B/T cells in the blood that expressed receptors for TSLP and IL-33, and that responded to these cytokines with rapid release of IL-5 and IL-13, in vitro. These cells were present in the sputum of allergic asthma patients, but not in the sputum of healthy individuals. More importantly, their numbers increased in response to specific allergen inhalation [75], suggesting a role of these ILC2-like cells in allergic asthma. However, it is unclear if these cells are the same as the Lin^−^ CD127^+^ CD161^+^ CRTH2^+^ ILC2s that were later characterized in humans [38].

Human ILC2s are present in both the naive and inflamed lung, and they have the ability to produce large amounts of type 2 cytokines after activation with IL-25, IL-33, or TSLP [11, 30]. Interestingly, it was shown that asthma patients have increased expression of IL-25, IL-33, and TSLP in the lungs [76–78]. Furthermore, genetic variants of TSLP, IL-17RB, IL-33, and ST2 have been associated with increased susceptibility to asthma [79–82]. Recently, several groups have investigated the role of ILC2s in the airways of asthmatics. Christianson et al. demonstrated an increased frequency of IL-13 producing ILC2s in BALF in comparison to disease control subjects. Interestingly, also the concentration of IL-33 was increased in BALF of asthmatics and correlated with disease severity [72]. In corticosteroid resistant severe asthma patients higher levels of ILC2s in induced sputum were observed compared with healthy controls but also with mild asthmatics. More importantly, ILC2s were the predominant source of IL-5 and IL-13, despite CD4^+^ T cells were more abundant [83]. Also in children with severe-therapy resistant-asthma, an increased number of ILC2s was found in induced sputum compared with children with lower respiratory tract infections without asthma [84]. It was shown before by Bartemes et al. that PBMCs from allergic asthma patients produced significantly larger amounts of IL-5 and IL-13 after treatment with IL-25 or IL-33 than PBMCs from allergic rhinitis patients or healthy controls. In addition, the total number of blood ILC2s and the proportion of ILC2s in PBMCs were significantly increased in allergic asthma patients compared to allergic rhinitis patients or healthy controls, suggesting that the enhanced type 2 response by PBMCs from allergic asthma patients might be caused by the increased presence of ILC2s [85]. In contrast, another study showed that ILC2s were present in similar numbers in the blood from healthy controls, mild allergic asthma patients, and severe allergic asthma patients [30]. However, an increased activation status of ILC2s during disease might still result in increased type 2 cytokine production or increased reactivity toward activating stimuli.

ILC2s have also been associated with chronic rhino-sinusitis (CRS). CRS is characterized by inflammation of the mucosal surfaces of the nose and para-nasal sinuses, and it often coexists with allergic asthma [86]. The presence of ILC2s was enriched in the nasal polyps of CRS patients compared to noninflamed nose tissue of CRS patients without nasal polyps or healthy controls [38, 87, 88]. Notably, CRS without nasal polyps and CRS with nasal polyps are typically associated with type 1 or type 2 inflammation, respectively [89]. Furthermore, it was shown that epithelial cells from CRS patients with nasal polyps had increased expression of TSLP and IL-33 [88, 90].

Recent studies showed that ILC2s are enriched in skin lesions of atopic dermatitis patients [36, 40]. Atopic dermatitis is a chronic inflammatory skin disease that is characterized by eosinophilic infiltration and high serum IgE levels [91]. Similar to allergic asthma and CRS, atopic dermatitis has been associated with increased expression of TSLP, IL-25, and IL-33 in the skin [92, 93]. ILC2s from skin lesions of atopic dermatitis patients showed increased expression of IL-17RB, ST2, and TSLPR compared to skin ILC2s from healthy controls, suggesting a role in the maintenance of the inflammation [40, 85]. Together, these studies indicate that ILC2s could be involved in several Th2-mediated disorders. In particular, ILC2s might be important key players in the pathogenesis of severe asthma for which there are limited treatment options. ILC2s might represent a potential therapeutic target.

## Conclusion and clinical implications

Allergic asthma is a complex disease that is mediated by various interactions between immune cells and structural cells (Fig. 1). Th2 cells are central effector cells in this process through their secretion of type 2 cytokines that drive IgE production and control the activity of various innate immune cells. The recently identified ILC2s are hypothesized to coordinate epithelial responses to allergen encounter and represent another major source of type 2 cytokines. Many studies support that ILC2s are involved in asthma pathogenesis by interacting with structural cells and the innate immune system. Importantly, recent studies indicate that ILC2s also amplify the adaptive type 2 response.

However, in order to fully assess their potential as a therapeutic target, some main questions remain to be answered. Firstly, the role of ILC2s in the immunopathology in human asthma needs to be further elucidated, as so far only associations have been found between increased presence of ILC2 or ILC2-activating stimuli and allergic disease. Secondly, the finding that ILC2s can induce eosinophil recruitment, mucus hyperproduction, and airway hyperresponsiveness in the absence of T and B cells in mice warrants further investigation of the relative contribution of ILC2s in comparison with the Th2-IgE-mast cell pathway to the development of shortness of breath, the most relevant clinical symptom in human asthma. In addition, the relative contribution of IL-5 and IL-13 production by ILC2s in human asthma in comparison to Th2 cells, other innate cells and structural cells, like epithelial cells [94], needs to be further elucidated. If it is proven that ILC2s contribute to the long-term exacerbations and chronic disease in allergic asthma, it remains an interesting challenge to determine how ILC2s should be targeted. Intervening with the activation of ILC2s might be difficult due to several other activation pathways that could bypass the blocked activation route. Indeed, only the combined blockage of IL-25, IL-33 and TSLP signaling could abolish ILC2 activation in response to chitin inhalation in mice [19]. Likewise, intervening with the type 2 effector cytokines that are provided by ILC2s might require inhibition of a combination of cytokines.

Importantly, direct intervention with ILC2 function requires further elucidation of their potential beneficial role in the respiratory system. ILC2s have been shown to promote tissue repair and lung homeostasis during influenza infection by secretion of amphiregulin. Interestingly, amphiregulin is an epidermal growth factor receptor (EGFR) ligand that has been linked to the regulation of tissue repair and remodeling in the context of acute asthma attacks and epithelial injury [95]. Inhibition of ILC2s during influenza or respiratory virus-induced asthma exacerbations may have both beneficial and detrimental effects [11, 22].

In conclusion, a remarkable amount of knowledge on ILC2 function has been gained since their discovery only six years ago. Murine studies suggest that these cells play an important role in the immunopathology of allergic asthma by interacting with both structural as innate immune and adaptive type 2 cells during allergen sensitization and acute inflammation. Now, the future challenges lie in further elucidation of both the pathogenic and protective functions of ILC2s in acute and chronic human allergic asthma, in order to better comprehend the complex pathogenesis of this disease and facilitate the design of novel therapeutics.
